# Perceptions and Experiences of Veterinary Assistants, Veterinary Technicians/Nurses, and Veterinary Technician Specialists

**DOI:** 10.1111/vec.70043

**Published:** 2025-10-21

**Authors:** Lori R. Kogan, Leslie Carter, Kelly Foltz

**Affiliations:** ^1^ Department of Clinical Sciences, College of Veterinary Medicine and Biomedical Sciences Colorado State University Fort Collins Colorado USA; ^2^ BluePearl Tampa Florida USA

**Keywords:** burnout, utilization, veterinary assistant, veterinary nurse, veterinary technician specialist, veterinary technician

## Abstract

**Objective:**

To examine the perceptions and experiences pertaining to the motivations, barriers, well‐being, and utilization of veterinary assistants and technicians/nurses (including veterinary technician specialists) and their influence on job fulfillment, burnout, and interest in remaining in the field.

**Design:**

Electronic survey distributed via veterinary organizations, associations, listservs, and social media from September 25, 2023, through January 7, 2024.

**Participants:**

Two thousand one hundred seventy‐six total participants.

**Setting and Interventions:**

Online survey.

**Measurement and Main Results:**

Participants included 153 veterinary assistants, 1600 veterinary technicians/nurses, and 414 veterinary technician specialists. Sixty‐four percent of respondents reported decreased interest in remaining in the field long term, with the most frequently endorsed factors for this change being inadequate pay, burnout/compassion fatigue, insufficient staff, and poor leadership. Respect at work, ability to contribute to animal well‐being, self‐improvement and professional development, and ability to effect change when identifying something that could be improved were significant predictors of burnout, fulfillment, and changed interest in remaining in the field long term.

**Conclusions:**

It is critically important to understand the lived experiences, causes of veterinary assistants’ and veterinary technicians/nurses’ burnout, and the resulting decreased interest in remaining in the field. Even with current shortages and financial limitations, many of the job characteristics identified in the current study can be changed within veterinary hospitals. The results from this study suggest that improvements to workplace culture should focus on respect, the power to effect change, ability to contribute to animal well‐being, and opportunities for professional development.

**Abbreviations**
AVMA: American Veterinary Medical Association
IPE: interprofessional education
DVM: veterinarian
NAVTA: National Association of Veterinary Technicians in America
PFI: Professional Fulfillment Index
VT/N: veterinary technician/nurse
VTS: veterinary technician specialist

## 1 Introduction

Veterinary technicians/nurses (VT/Ns) are instrumental in providing optimal patient care and practice efficiency, with one additional credentialed VT/N estimated to increase practice revenue by approximately 20.5% [1, 2, 3, 4]. Although the current ratio of VT/Ns to veterinarians in companion animal practices is approximately 2:1, it has been suggested this ratio should be closer to 10:1 for ideal revenue and productivity [2].

In 2019, there were an estimated 118,000 VT/Ns in the United States [5]. Although the exact number of VT/Ns who are credentialed is unknown, an estimate in 2017 placed this number at 70,000 [5]. In addition, there are far fewer veterinary technician specialists (VTS), with estimates of 1100 in 2017 [5] and 1511 in 2024 [6]. Over the last 6 years, the number of students sitting annually for the Veterinary Technician National Examination has been around 7000, far short of the number needed to fill the current needs [7]. To address this shortage, it has been suggested that increasing the number of accredited VT/N educational programs in the United States be prioritized and enrollment increased at existing programs [7]. This approach, however, does not address the retention of credentialed VT/Ns and VTSs or the attrition from the profession. It is possible that, despite the projected positive job market, current challenges within the industry are negatively affecting the number of people who decide to pursue a career as a credentialed VT/N or remain in the field long term.

Burnout (feelings of emotional depletion or exhaustion, depersonalization, and reduced personal accomplishment) [8] and compassion fatigue (vicarious or secondary trauma) [9] can lead to serious negative physical and psychological impairment and are highly prevalent among VT/Ns [10, 11]. Studies suggest that more than 50% of VT/Ns struggle with feelings of burnout or compassion fatigue [12, 13, 14]. Numerous studies have explored burnout among both veterinarians and VT/Ns and have identified contributing work factors, including excessive workloads, long work hours, on‐call duties, limited resources, workplace conflicts, euthanasia, unrealistic expectations from pet owners, moral injury, and high debt load [13, 15, 16, 17, 18, 19, 20, 21]. In contrast, factors that appear to mitigate burnout include positive workplace culture, feelings of autonomy and control, feeling like one is part of a team, social support and respect, opportunities for professional development, and the ability to develop and use one's professional skills [16, 18, 22, 23, 24, 25, 26, 27].

To help address the shortage of VT/Ns, it is critically important to better understand the factors that predict burnout, job fulfillment, and a desire to remain in the field long term. The current study was designed to help answer these questions by gathering information to better understand the perceptions and experiences of VT/Ns and the factors that may improve their work experience.

## 2 Materials and Methods

This study was reviewed and classified as exempt by the Colorado State University Institutional Review Board. An anonymous online survey (Appendix S1) was created with commercial survey software^1^ to assess the perceptions and experiences of currently practicing veterinary assistants and VT/Ns (including VTS, who are credentialed VT/Ns with additional VTS credentials). The survey was distributed through veterinary‐related listservs, including the Veterinary Emergency and Critical Care Society, the Academy of Veterinary Emergency and Critical Care Technicians and Nurses, 15 additional VTS academies, the Veterinary Nurses Council of Australia, the American College of Veterinary Emergency & Critical Care, the National Association of Veterinary Technicians in America (NAVTA), and veterinary social media sites (see the Appendix for full list). The survey, made available from September 25, 2023, through January 7, 2024, was voluntary, and participants could choose to stop at any point. Participants could also choose to skip any questions they did not wish to answer.

Demographic questions included role, credential status, supervisory role, current place of employment, structure of the practice's ownership, degree, time in the field, age, sex, race, ethnicity, and country; where applicable, participants could select multiple answers (e.g., for credentials held). Participants were presented with a series of items to indicate if and how veterinary staff job titles, roles, and tasks are differentiated by credential. Questions were included that pertained to utilization levels, reasons for lack of full utilization, and obstacles to learning new skills. Next, the participant's interest in the field as a long‐term profession was assessed, both upon entry to the field and at the time of survey completion. They were also asked to provide reasons for any decreased interest in the field.

Burnout and professional fulfillment were assessed with the Stanford Professional Fulfillment Index (PFI) [28], which consists of 16 items and three scales. Two of the three scales measure burnout, either through work exhaustion or by interpersonal disengagement, and professional fulfillment is measured by the third scale. Responses to professional fulfillment items ranged from 0 = *Not at all true* to 4 = *Completely true*, and responses to work exhaustion and interpersonal disengagement items ranged from 0 = *Not at all* to 4 = *Extremely*. Each scale was treated as a continuous variable, with scale scores calculated by averaging the item scores. Higher scores on the professional fulfillment scale indicate a higher level of fulfillment; likewise, higher scores on the work exhaustion or interpersonal disengagement scales indicate higher levels of work exhaustion and interpersonal disengagement. Dichotomous burnout classification is calculated using an average item score of all 10 burnout items (work exhaustion and interpersonal disengagement) with a cutoff score of 1.33 [28]. Dichotomous professional fulfillment classification is calculated using an average item cutoff score of ≥3.0 [28].

### 2.1 Statistical Analyses

Descriptive statistics were calculated for most questions. Differences in responses based on various demographic factors were examined with *χ* ^2^ tests (after checking for violations of assumptions). The relationship between multiple variables and burnout, fulfillment, and decreased interest in remaining in the profession long term was assessed with the Pearson correlation test. These variables included demographics (supervisory role [yes/no], role [veterinary assistant, uncredentialed VT/N, VT/N credentialed, VTS], time in the field [≤5, 6–10, 11–15, 16–20, 21–30, >30 years], type of workplace [emergency and specialty, emergency only, general practice and emergency, other], and structure of the practice's ownership [corporate, private]) as well as the 10 most important job characteristics. Significant correlations of ≥0.200 were used as predictor variables in multiple linear regression (burnout and fulfillment) and ordinal regression (changed interest in the field long term). All analyses were performed with commercial statistical software^2^. Statistical significance was set at *p* < 0.05.

## 3 Results

### 3.1 Demographics

A total of 2176 veterinary assistants, VT/Ns (excluding VTS; not credentialed: 222 [9%], credentialed: 1378 [56%]), and VTS (414 [17%]), primarily identifying as White (1503/1776 [85%]), female (1580/1761 [89%]), and working in the United States (1637/1778 [92%]), completed the survey. The majority of participants reported working in a clinical setting (1554/2101 [74%]), and of these, 72% (1024/1536) reported working at a corporate‐owned hospital. Participants’ time in the field ranged from ≤5 years (323/1778 [18%]) to >30 years (104/1778 [6%]). The participants’ ages ranged from 18–29 years (413/1773 [23%]) to ≥50 years (220/1773 [12%]). Most participants reported they were not in a supervisory position (1409/2171 [65%]). Nearly all participants reported having a degree beyond high school (1915/2154 [89%]). VTS (*n* = 414) were asked to indicate their specialty, with the largest proportion reporting being members of the Academy of Veterinary Emergency & Critical Care Technicians and Nurses (212 [51%]) (Table S1).

### 3.2 Credentialing

Credentialed VT/Ns (excluding VTS) (*n* = 1378) were asked to indicate their specific credentials, the most common of which were certified VN (596 [43%]), registered VN (509 [37%]), and licensed VN (329 [24%]). VT/Ns who identified as not being credentialed (14% of VT/Ns, *n* = 222) were asked to indicate the reasons why they were not. The most commonly reported reasons included cost (144 [65%]), respondents did not think it would lead to a substantial improvement in pay (130 [59%]), time investment required (99 [45%]), respondents’ disbelief that it would lead to a difference in job duties (93 [42%]), and questioning the value of becoming credentialed (72 [32%]).

### 3.3 Role Differentiation

When participants were asked if the roles of veterinary staff at their place of employment were differentiated based on credentials (*n* = 2049), 815 (40%) responded “always,” 465 (23%) responded “most of the time,” 421 (21%) responded “sometimes,” and 348 (17%) responded “no.” For those who indicated that roles are differentiated by credentials (*n* = 1701), the most commonly reported methods (respondents could select >1) for differentiation included name badge (1173 [69%]), schedule (780 [46%]), and uniform marking (672 [40%]). Participants were then asked if the tasks veterinary staff are asked to perform differ by credentials (*n* = 2052), to which 283 (14%) replied “nearly always,” 378 (18%) replied “most of the time,” 838 (41%) replied “sometimes,” and 553 (27%) replied “no, it does not seem to matter.”

### 3.4 Skill and Knowledge Utilization

Participants were asked to indicate the percentage of their skills and knowledge they feel are being utilized (*n* = 2010). A total of 517 (26%) reported ≤50%, 607 (30%) reported 51%–70%, 608 (30%) reported 71%–90%, and 278 (14%) reported that >90% of their skills and knowledge are utilized. The difference was significant when the percentage of skills and knowledge utilized was assessed by role (*p* = 0.022). Veterinary assistants (46 [32%]) and VTS (128 [33%]) were more likely to report ≤50% of their skills were utilized than credentialed VT/Ns (298 [23%]) or VT/Ns not credentialed (45 [23%]).

Participants who reported feeling that ≤90% of their skills and knowledge were utilized were asked to indicate their perceptions of why this was the case (*n* = 1732). The most common reasons included “culture of the workplace limits tasks techs/nurses are allowed to perform” (633 [37%]), “Veterinarians’ [DVMs’] lack of trust” (594 [34%]), “DVMs are unaware of my skill/training level” (562 [32%]), “DVMs don't perceive techs/nurses as capable of performing advanced skills” (520 [30%]), and “DVMs prefer to do tasks themselves” (519 [30%]).

Participants were asked to estimate the percentage of time they spend performing tasks that could be performed by staff with less training or skills (*n* = 1959). A total of 176 (9%) reported ≤10%, 571 (29%) reported 11%–30%, 667 (34%) reported 31%–50%, and 545 (28%) reported >50% of their time was spent performing tasks that could be performed by colleagues with less training or skills. When the percentage of time spent performing tasks that could be performed by staff with less skill/training was assessed by role, there was a significant difference (*p* < 0.001). VT/Ns, whether credentialed or not, and VTS reported a larger percentage of their time spent performing tasks that could be performed by staff with less skill or training than veterinary assistants.

When asked if there are skills they could legally perform but lacked the training or knowledge to do so (*n* = 2000), 1212 (61%) of participants said “yes.” When asked to indicate the obstacles they felt impeded their ability to learn new skills, lack of dedicated training time (818 [67%]), lack of mentorship (565 [47%]), lack of agreement about who should perform the skill (472 [39%]), and lack of a training program (450 [37%]) were the most common responses.

### 3.5 Client Education

Participants were asked if their place of employment educates clients about the credentials, education, and role of VT/Ns (*n* = 1960), to which 434 (22%) responded “yes.” The most common methods of educating clients about their roles included the practice's website or social media accounts (268 [62%]), word of mouth (261 [60%]), and hospital posters (130 [30%]).

### 3.6 Training

Participants were asked their opinions regarding a training model for VT/Ns new to the field of emergency medicine to help prepare them for work in the specialty (*n* = 1738). Most participants (1070 [62%]) felt it would have a positive effect, 102 (6%) felt it would have no influence, 45 (3%) felt it would have a negative effect, and 521 (30%) felt they would need more information about the proposed training model. There was a significant difference when responses were assessed by role (*p* < 0.001), with VTS more likely to report a training model would have a negative effect (11%) versus a positive effect (54%). Veterinary assistants expressed the inverse, with more emphasis on the positive effects of a training model (negative: 1 [1%]; positive: 75 [66%]). Likewise, VT/Ns not credentialed (negative: 1 [1%]; positive: 102 [63%]) and credentialed VT/Ns (negative: 3 [<1%]; positive: 696 [63%]) both viewed a training model as having a positive effect.

### 3.7 Interest in a Long‐Term Profession as a VT/N

Upon entering the field (*n* = 2043), most participants indicated being extremely (1548 [76%]) or somewhat (335 [16%]) interested in the field as a long‐term profession. Seven percent (*n* = 146) of participants reported minimal or no interest, and 14 (1%) did not remember how they felt when entering the field. When asked if their interest in being a VT/N as a long‐term profession had changed over time (*n* = 2031), 288 (14%) reported their interest had increased slightly (119 [6%]) or significantly (169 [8%]), while 450 (22%) reported no change, 769 (38%) reported it had declined slightly, and 524 (26%) reported it had declined significantly. A significant relationship was found between participants’ change of interest in their position as a long‐term profession and their amount of time in the field. Those in the field between 6 and 15 years were more likely to report a decreased interest (70%) than those in the field ≤5 (52%), 16–20 (61%), 21–30 (62%), or >30 years (48%) (*p* < 0.001).

Participants who indicated a decreased interest in the field as a long‐term profession were asked to rate how several work factors influenced their change in interest. The factors reported as having the most significant negative impact included inadequate pay (significant negative impact: 824/1248 [66%]), burnout/compassion fatigue (significant negative impact: 683/1250 [66%]), insufficient staff for optimal patient care (significant negative impact: 646/1247 [52%]), poor leadership (significant negative impact: 613/1236 [50%]), and a negative/toxic workplace environment (significant negative impact: 580/1235 [47%]) (Table 1).

**TABLE 1:** Reasons given by 2176 veterinary assistants, technicians/nurses, and technician specialists for a decreased interest in remaining in the field long term.

	Minimal/no negative impact		Moderate negative impact		Significant negative impact	
	*N*	%	*N*	%	*N*	%
Inadequate pay/salary (*n* = 1248)	73	6%	351	28%	824	66%
Burnout/compassion fatigue/moral injury (*n* = 1250)	138	11%	429	34%	683	55%
Insufficient staff for optimal patient care (*n* = 1247)	141	11%	460	37%	646	52%
Poor leadership (*n* = 1236)	197	16%	426	35%	613	50%
Negative/toxic workplace environment (*n* = 1235)	212	17%	443	36%	580	47%
Lack of acknowledgment/appreciation (*n* = 1237)	238	19%	467	38%	532	43%
Lack of opportunity for advancement (*n* = 1223)	293	24%	438	36%	492	40%
Unable to drive effective change or improvement at work (*n* = 1225)	242	20%	498	41%	485	40%
Physical demands of the job (*n* = 1245)	409	33%	436	35%	400	32%
Lack of mentoring/training/support (*n* = 1225)	365	30%	476	39%	384	31%
Few professional development opportunities (*n* = 1203)	381	32%	444	37%	378	31%
Underutilization (*n* = 1169)	449	38%	361	31%	359	31%
Competition with uncredentialed staff (*n* = 1145)	486	42%	308	27%	351	31%
Disrespect/incivility in the workplace (*n* = 1194)	414	35%	430	36%	350	29%
Lack of control over one's schedule (days/hours) (*n* = 1187)	514	43%	388	33%	285	24%
Shift length (*n* = 1216)	684	56%	362	30%	170	14%
Requirement to work overnight shifts (*n* = 812)	515	63%	144	18%	153	19%

*Note*: Participants who reported decreased interest in being a VT/N as a long‐term profession (*n* = 1293) were asked to rate the level of impact of 17 work factors on their decreased interest in order of most to least significant negative impact.

Abbreviation: VT/N, veterinary technician/nurse.

### 3.8 Satisfaction and Importance of Job Characteristics

Participants were asked to rate their satisfaction with 24 different job characteristics. Those characteristics offering the highest levels of satisfaction were the ability to contribute to animal well‐being (1461/1899 [77%]), case variety (1231/1897 [65%]), sense of competency (1183/1895 [62%]), being a part of a team with an important mission (1090/1898 [57%]), length of shifts (1075/1900 [57%]), feeling that veterinarians are aware of one's skills (1066/1898 [56%]), and intellectual stimulation (989/1899 [52%]) (Table 2).

**TABLE 2:** Satisfaction level of 2176 veterinary assistants, technicians/nurses, and technician specialists with 24 job characteristics.

	Unsatisfied		Neutral		Satisfied	
	*N*	%	*N*	%	*N*	%
Ability to contribute to animal well‐being (*n* = 1899)	166	9%	272	14%	1461	77%
Variety of cases (*n* = 1897)	212	11%	454	24%	1231	65%
Sense of competency (*n* = 1895)	254	13%	460	24%	1183	62%
Being a part of a team with an important mission (*n* = 1898)	338	18%	470	25%	1090	57%
Length of shifts (*n* = 1900)	257	14%	568	30%	1075	57%
The feeling that the veterinarians one works with are aware of one's skills (*n* = 1898)	364	19%	468	25%	1066	56%
Intellectual stimulation (*n* = 1899)	427	23%	483	25%	989	52%
The feeling of adding value to the practice or place of employment (*n* = 1900)	462	24%	493	26%	945	50%
Respect at work (*n* = 1900)	480	25%	491	26%	929	49%
Control over one's schedule (days/hours) (*n* = 1899)	429	23%	547	29%	923	49%
Flexibility of schedule (*n* = 1899)	440	23%	567	30%	892	47%
Appropriate utilization of one's professional skills (*n* = 1899)	560	30%	472	25%	867	46%
Sense of autonomy (*n* = 1898)	361	19%	683	36%	854	45%
Self‐improvement or professional development (*n* = 1900)	501	26%	596	31%	803	42%
Workplace environment (*n* = 1899)	548	29%	596	31%	755	40%
Physical demands of the job (*n* = 1899)	471	25%	720	38%	708	37%
Support from leadership (*n* = 1899)	758	40%	485	26%	656	35%
Amount of acknowledgment/appreciation (*n* = 1899)	746	39%	519	27%	634	33%
Leadership (*n* = 1900)	706	37%	563	30%	631	33%
Mentoring (*n* = 1899)	698	37%	626	33%	575	30%
The ability to effect change when one sees something that could be improved (*n* = 1900)	898	47%	440	23%	562	30%
Ability for career mobility (*n* = 1898)	709	37%	648	34%	541	29%
Pay/salary (*n* = 1898)	1020	54%	372	20%	506	27%
Amount/number of staff for optimal patient care (*n* = 1899)	1047	55%	418	22%	434	23%

*Note*: All participants were asked to rate their satisfaction with 24 different job characteristics. Responses are arranged in order of greatest percentage satisfied.

Differences in satisfaction level by job role (veterinary assistant, VT/N not credentialed, VT/N credentialed, VTS) were also assessed (Table 3). Participants were asked to indicate the importance of the same 24 different job characteristics. The factors reported as most important for job satisfaction were respect at work (1714/1820 [94%]), ability to contribute to animal well‐being (1713/1819 [94%]), pay (1706/1820 [94%]), and appropriate utilization of professional skills (1702/1820 [94%]) (Table 4).

**TABLE 3:** Satisfaction of 2176 veterinary assistants, technicians/nurses, and technician specialists with 24 job characteristics by job role.

	Veterinary assistant		Veterinary technician not credentialed		Veterinary technician credentialed		Veterinary technician specialist	
	*N*	%	*N*	%	*N*	%	*N*	%
**Pay/salary (*χ* ^2^** = **87.71 (6), *p* < 0.001)** **VTS higher than VA and VTNC; VTC higher than VA and VTNC**	**15**	**12%**	**28**	**15%**	**311**	**26%**	**152**	**40%**
One's ability to contribute to animal well‐being (*χ* ^2^ = 9.30 (6), *p* = 0.157)	97	75%	140	77%	949	79%	275	72%
**Sense of competency (*χ* ^2^** = **23.45 (6), *p* < 0.001)** **VTS and VTC higher than VTNL and VA**	**70**	**55%**	**91**	**50%**	**787**	**65%**	**235**	**62%**
**Variety of cases (*χ* ^2^** = **32.23 (6), *p* < 0.001)** **VTS lower than VA, VTNC, and VTC**	**88**	**68%**	**133**	**74%**	**808**	**67%**	**202**	**53%**
Intellectual stimulation (*χ* ^2^ = 5.13 (6), *p* = 0.527)	64	50%	96	53%	641	53%	188	49%
Being a part of a team with an important mission (*χ* ^2^ = 3.39 (6), *p* = 0.759)	73	57%	95	52%	702	58%	220	58%
**Amount/number of staff for optimal patient care (*χ* ^2^** = **17.93 (6), *p*** = **0.006)** **VTS higher than VA, VTNC, and VTC**	**24**	**19%**	**27**	**15%**	**275**	**23%**	**108**	**28%**
**Workplace environment (*χ* ^2^** = **22.19 (6), *p* < 0.001)** **VTNC lower than VA, VTC, and VTS**	**55**	**43%**	**60**	**33%**	**500**	**41%**	**140**	**37%**
Leadership (*χ* ^2^ = 11.16 (6), *p* = 0.084)	43	33%	49	27%	407	34%	132	35%
**Mentoring (*χ* ^2^** = **21.29 (6), *p*** = **0.002)** **VTS higher than VTNC and VTC**	**45**	**35%**	**48**	**26%**	**344**	**29%**	**138**	**36%**
Physical demands of the job (*χ* ^2^ = 2.88 (6), *p* = 0.823)	51	40%	67	37%	455	38%	135	36%
**Amount of acknowledgment/appreciation (*χ* ^2^** = **21.89 (6), *p* < 0.001)** **VTS higher than VA, VTNC, and VTC**	**42**	**33%**	**47**	**26%**	**384**	**32%**	**161**	**42%**
Flexibility of schedule (*χ* ^2^ = 6.05 (6), *p* = 0.417)	56	43%	72	40%	579	48%	185	49%
**Sense of autonomy (*χ* ^2^** = **33.78 (6), *p* < 0.001)** **VTS higher than VA and VTNL; VTC higher than VTNC and VA**	**39**	**30%**	**65**	**36%**	**549**	**46%**	**201**	**53%**
Control over one's schedule (days/hours) (*χ* ^2^ = 5.35 (6), *p* = 0.500)	64	50%	76	42%	596	49%	187	49%
Length of one's shifts (*χ* ^2^ = 5.44 (6), *p* = 0.489)	70	54%	96	53%	698	58%	211	55%
Appropriate utilization of one's professional skills (*χ* ^2^ = 7.42 (6), *p* = 0.284)	51	40%	83	46%	551	46%	182	48%
**Respect at work (*χ* ^2^** = **15.65 (6), *p*** = **0.016)** **VTS higher than VA and VTNC**	**59**	**46%**	**74**	**41%**	**588**	**49%**	**208**	**55%**
Support from leadership (*χ* ^2^ = 10.76 (6), *p* = 0.096)	52	40%	51	28%	412	34%	141	37%
The feeling of adding value to the practice or place of employment (*χ* ^2^ = 6.90 (6), *p* = 0.330)	61	47%	77	42%	603	50%	204	54%
The feeling that the veterinarians one works with are aware of one's skills (*χ* ^2^ = 11.89 (6), *p* = 0.065)	61	47%	96	53%	705	58%	204	54%
**Ability to effect change when one sees something that could be improved (*χ* ^2^** = **25.85 (6), *p* < 0.001)** **VTS higher than VA, VTNC, and VTC; VTC higher than VA and VTNC**	**32**	**25%**	**39**	**21%**	**346**	**29%**	**145**	**38%**
Ability for career mobility (*χ* ^2^ = 5.62 (6), *p* = 0.467)	37	29%	48	26%	332	28%	124	33%
**Self‐improvement or professional development (*χ* ^2^** = **22.60 (6), *p* < 0.001)** **VTS higher than VA, VTNC, and VTC; VTC higher than VA**	**42**	**33%**	**67**	**37%**	**501**	**42%**	**193**	**51%**

*Note*: All participants were asked to rate their satisfaction with 24 different job characteristics. Responses are arranged in order of greatest percentage satisfied. Bold text denotes statistical significance. Numbers in parentheses indicate degrees of freedom.

Abbreviations: VA, veterinary assistant; VTC, veterinary technician credentialed; VTNC, veterinary technician not credentialed; VTS, veterinary technician specialist.

**TABLE 4:** Importance of 24 job characteristics reported by 2176 veterinary assistants, technicians/nurses, and technician specialists.

	Unimportant		Neutral		Important	
	*N*	%	*N*	%	*N*	%
Respect at work (*n* = 1820)	29	2%	77	4%	1714	94%
One's ability to contribute to animal well‐being (*n* = 1819)	36	2%	70	4%	1713	94%
Pay/salary (*n* = 1820)	50	3%	64	4%	1706	94%
Appropriate utilization of one's professional skills (*n* = 1820)	41	2%	77	4%	1702	94%
Sense of competency (*n* = 1820)	36	2%	94	5%	1690	93%
Support from leadership (*n* = 1820)	44	2%	92	5%	1684	93%
Workplace environment (*n* = 1820)	32	2%	113	6%	1675	92%
Amount/number of staff for optimal patient care (*n* = 1819)	40	2%	118	7%	1661	91%
Self‐improvement or professional development (*n* = 1818)	41	2%	127	7%	1650	91%
The ability to effect change when one sees something that could be improved (*n* = 1820)	41	2%	129	7%	1650	91%
Intellectual stimulation (*n* = 1820)	37	2%	137	8%	1646	90%
The feeling that the veterinarians one works with are aware of one's skills (*n* = 1819)	52	3%	125	7%	1642	90%
The feeling of adding value to the practice or place of employment (*n* = 1820)	49	3%	150	8%	1621	89%
Leadership (*n* = 1820)	37	2%	172	10%	1611	89%
Being a part of a team with an important mission (*n* = 1820)	56	3%	175	10%	1589	87%
Amount of acknowledgment/appreciation (*n* = 1820)	62	3%	219	12%	1539	85%
Sense of autonomy (*n* = 1817)	42	2%	268	15%	1507	83%
Mentoring (*n* = 1819)	59	3%	296	16%	1464	81%
Flexibility of schedule (*n* = 1820)	49	3%	333	18%	1438	79%
Control over one's schedule (days/hours) (*n* = 1820)	61	3%	334	18%	1425	78%
Ability for career mobility (*n* = 1820)	60	3%	337	19%	1423	78%
Variety of cases (*n* = 1818)	69	4%	436	24%	1313	72%
Length of shifts (*n* = 1819)	116	6%	520	29%	1183	65%
Physical demands of the job (*n* = 1820)	101	6%	608	33%	1111	61%

*Note*: All participants were asked to rate the importance of 24 different job characteristics. Responses are arranged in order of greatest importance.

### 3.9 Job Burnout and Fulfillment

Job burnout was assessed by averaging the 10 items related to burnout (work exhaustion and interpersonal disengagement) on the Stanford PFI [28]. A cutoff of 1.33 has been recommended for dichotomous burnout categories. A total of 1100 of 1784 (61.7%) participants fell above the cutoff for burnout. There was no difference based on job role (veterinary assistant, VT/N not credentialed, VT/N credentialed, VTS) (*p* = 0.131).

Job fulfillment was assessed by averaging the six questions of the PFI, with dichotomous professional fulfillment categories determined using the cutoff score of >3.0. Three hundred sixty‐eight of 1784 (20.6%) participants scored higher than the cutoff score for fulfillment. A significant difference was found between job roles (veterinary assistant, VT/N not credentialed, VT/N credentialed, VTS) (*p* = 0.037), with Bonferroni post hoc analysis indicating a difference between veterinary assistants (*χ* ^2^ = 2.01) and VTS (*χ* ^2^ = 2.26).

### 3.10 Multiple Linear Regression: Burnout

Pearson correlations between participant demographics (supervisory role [yes/no], role [veterinary assistant, VT/N not credentialed, VT/N credentialed, VTS], time in the field [≤5, 6–10, 11–15, 16–20, 21–30, >30 years], type of workplace [emergency and specialty, emergency only, general practice and emergency, other], and structure of the practice's ownership [corporate, private]) and satisfaction level with the top 10 important job characteristics were performed with burnout to identify potential predictors for the regression model. No demographics were significantly correlated at ≥0.10, and therefore, none were included in the model. All of the top 10 important job characteristics were significantly correlated at ≥0.10 and therefore entered into the model (Table S2). The multiple linear regression for burnout was significant (*F* = 65.03, *p* < 0.001; *r* ^2^ = 0.269). The significant predictors included satisfaction with respect (*p* > 0.001), ability to contribute to animal well‐being (*p* > 0.001), pay (*p* = 0.023), workplace environment (*p* > 0.001), amount of staff for optimal patient care (*p* > 0.001), self‐improvement or professional development (*p* = 0.002), and ability to effect change when identifying something that could be improved (*p* = 0.032). The strongest predictors were the ability to contribute to animal well‐being and workplace environment (Table S3).

### 3.11 Multiple Linear Regression: Fulfillment

Pearson correlations between participant demographics (supervisory role [yes/no], role [veterinary assistant, VT/N not credentialed, VT/N credentialed, VTS], time in the field [≤5, 6–10, 11–15, 16–20, 21–30, >30 years], type of workplace [emergency and specialty, emergency only, general practice and emergency, other], and structure of the practice's ownership [corporate, private]) and satisfaction level with the top 10 important job characteristics were performed with fulfillment to identify potential predictors for the regression model. Supervisory role was the only demographic correlated at ≥0.10 (*r* = 0.165) and therefore the only demographic included in the model. All of the top 10 important job characteristics were significantly correlated at ≥0.10 and were entered into the model (Table S4). The multiple linear regression for fulfillment was significant (*F* = 175.10, *p* < 0.001; *r* ^2^ = 0.521). The significant predictors included supervisory role (*p* = 0.012), satisfaction with respect (*p* > 0.001), ability to contribute to animal well‐being (*p* > 0.001), appropriate utilization of professional skills (*p* > 0.001), sense of competency (*p* > 0.001), workplace environment (*p* > 0.001), self‐improvement or professional development (*p* > 0.001), and ability to effect change when identifying something that could be improved (*p* > 0.001). The strongest predictors were the ability to contribute to animal well‐being and workplace environment (Table S5).

### 3.12 Ordinal Regression: Changed Interest in Job as a Long‐Term Career

Pearson correlations between participant demographics (supervisory role [yes/no], role [veterinary assistant, VT/N not credentialed, VT/N credentialed, VTS], time in the field [≤5, 6–10, 11–15, 16–20, 21–30, >30 years], type of workplace [emergency and specialty, emergency only, general practice and emergency, other], and structure of the practice's ownership [corporate, private]) and satisfaction level with the top 10 important job characteristics were performed with a change in interest in the field as a long‐term profession to identify potential predictors for the regression model. No demographics were significantly correlated at ≥0.10 and were therefore not included in the model. All of the top 10 important job characteristics were significantly correlated at ≥0.10 and were entered into the model (Table S6). The ordinal regression for changed interest in the field was significant (*χ* ^2^ = 326.26 (10), *p* < 0.001; *r* ^2^ = 0.190). The significant predictors included satisfaction with respect (*p* = 0.022), ability to contribute to animal well‐being (*p* = 0.022), pay (*p* > 0.001), number of staff for optimal patient care (*p* = 0.008), self‐improvement or professional development (*p* = 0.010), and ability to effect change when identifying something that could be improved (*p* = 0.005) (Table S7).

### 3.13 Common Predictors: Burnout, Fulfillment, and Changed Interest in the Field as a Long‐Term Profession

Satisfaction with four of the top 10 important job characteristics was a significant predictor of burnout, fulfillment, and changed interest in the field as a long‐term profession: respect at work, ability to contribute to animal well‐being, self‐improvement or professional development, and ability to effect change when identifying something that could be improved. An additional three items predicted two of these factors. Pay and number of staff for optimal patient care were significant predictors for burnout and changed interest in the field. Workplace environment was a significant predictor of both burnout and fulfillment. Appropriate utilization of professional skills and sense of competency were only significant predictors for fulfillment. Support from leadership was not a significant predictor for burnout, fulfillment, or changed interest in remaining in the field long term.

## 4 Discussion

The results of our study found that 61% of participants reported feeling burned out, similar to recent reports of burnout in 50% of veterinarians and 58% of VT/Ns [13, 29]. The strongest predictors of burnout for VT/Ns included satisfaction levels with respect, ability to contribute to animal well‐being, and workplace environment. Personal factors such as role, time in the field, type of workplace, or private versus corporate setting were not significant predictors of burnout. Respect and workplace environment include the dynamics between VT/Ns and veterinarians. Research with physicians and nurses suggests that positive collaborative relationships that include joint decision‐making and responsibility sharing lead to higher nurse satisfaction and retention [30, 31, 32, 33].

The strongest predictors of job fulfillment were respect, ability to contribute to animal well‐being, and workplace environment; only 21% of VT/Ns met or exceeded the cutoff for this item. This percentage is similar to the 22% of veterinarians reported to have high levels of compassion satisfaction (the pleasure one derives from being able to help their patients, also characterized as the positive side of caregiving) [12, 29]. In our study, job fulfillment was significantly higher for VTS (24%) than for veterinary assistants (15%). VTS scores were not significantly higher than VT/Ns (credentialed or not [20%]).

When asked if their interest in being a VT/N as a long‐term profession had changed, 64% of participants reported it had declined over time. This percentage is higher than the percentage of veterinarians in private practice who have reported considering leaving the field before retirement (44%) [29]. The strongest predictors of this decline in interest were pay, ability to contribute to animal well‐being, self‐improvement or professional development, and ability to effect change.

The findings from our study suggest that systemic issues rather than personal ones may predict burnout, job fulfillment, and intent to remain in the field, yet many veterinary workplace interventions have focused on personal well‐being [17]. Although personal well‐being programs are helpful for some individuals [34], a focus on individual solutions without attention to organizational and situational factors is likely to have minimal effects [35].

One important element of VT/Ns’ ability to contribute to animal well‐being is utilization of education and clinical skills [13]. Our study found that only 14% of participants felt that most (≥90%) of their skills were utilized. Allowing more VT/Ns to perform at their skill level is not only important to VT/Ns but is one of the American Veterinary Medical Association's (AVMA) recommendations to help address the shortage of veterinarians [36].

Twenty‐eight percent of participants reported that >50% of their time was spent performing tasks that could be performed by staff with less training or skills. When queried as to why their skills are not better utilized, the primary reasons included workplace culture, veterinarians’ lack of trust, and veterinarians’ being unaware of their skill or training level. Similar findings were reported in the recent NAVTA report in which 54% of participants indicated feeling that credentialed VT/Ns and veterinary assistants are trained to perform the same job duties, and 20% reported feeling they are not utilized to their fullest potential [14]. In the NAVTA study, primary barriers were similar to those identified in our study and included veterinarians’ lack of trust and unwillingness to let VT/Ns complete tasks they are trained to perform, along with veterinary assistants that are permitted to perform VT/Ns’ tasks. This may relate directly to underutilization and issues of clinician trust cited by our participants. The longstanding practice of “title creep,” in which veterinary assistants are misidentified as VT/Ns and trained to do the work of a credentialed VT/N, creates a situation in which veterinarians are unable to rely on consistent education, knowledge, and clinical skills within the labor pool when sourcing nondoctor labor. “Title creep” may also contribute to VT/N disengagement and lower wages across the industry. Despite the fact that these barriers could be addressed with training and education, 20% of VT/Ns reported that nothing is being done at their hospitals to improve utilization of their complete skill set [14]. Providing opportunities for VT/Ns to demonstrate their skills or teach veterinary assistants might be another innovative way to help improve utilization. Future research to better understand veterinarians’ perceptions pertaining to utilization would be helpful. In addition to the ability to use one's professional skills, feeling like one is part of a team is a critical component in VT/N job fulfillment [16, 18, 22, 23, 24, 25, 26, 27]. Given the important role played by VT/Ns in practice revenue and veterinarian productivity [2], creating a supportive team environment is a critical component not only to the well‐being of veterinary professionals but to the financial success of the practice. “Respect” and its variants were repeatedly selected by survey participants as determining factors in their professional longevity and satisfaction.

Although 86% of the VT/Ns in our survey (excluding VTS) were credentialed, when asked if the tasks veterinary staff are asked to perform in their hospital differ by credentials, 41% reported “only sometimes,” and 27% said “no,” that it does not matter. This is troubling but not necessarily surprising, considering the time and financial sacrifice entailed with credentialing. It also explains why when those not credentialed were asked to indicate the barriers, their primary reasons included cost (38%), disbelief that it would lead to substantial improvement in pay, and disbelief that it would lead to a difference in job duties. The results of our study appear to validate these concerns, and attempting to add more VT/N students to the pipeline will not fix the shortage if the field does not address the underlying issues of clinician education surrounding VT/N, VTS skill sets, title protection, retention, and attrition. Clarifying nomenclature by educating all hospital staff on the different skills and knowledge credentialed VT/Ns have compared with veterinary assistants and VTS, as well as creating a system to easily identify all roles within the practice (e.g., nametags, colored smocks, publicly posted degrees and credentials, etc.), can help veterinarians best utilize each staff member. It is also important to ensure remuneration and that daily job duties are appropriately differentiated.

Successful systemic changes must include both intrinsic and extrinsic job factors [37]. Intrinsic rewards found to mitigate burnout for VT/Ns include control over one's work schedule, opportunities to learn, a sense of respect from colleagues, and the ability to use one's skills and knowledge [13]. Better utilization and respect for the knowledge and expertise of each team member can not only help improve job satisfaction among VT/Ns but will simultaneously help the field meet the growing demand for veterinary care [36]. Allowing VT/Ns to use their skills and knowledge and providing opportunities for learning (including designated protected time and structured mentoring) will likely positively increase VT/Ns’ sense of accomplishment and engagement, helping them feel that their work is meaningful and fulfilling [23, 38, 39].

One less commonly discussed element of technician utilization is the role of veterinarians, including veterinary students, in the overall promotion of the VT/N profession. As comments from this survey have shown, clinician trust and understanding are integral to VT/N retention and utilization. Participants indicated that it would be optimal for veterinarians to better understand the education, clinical skills, and credentialing requirements for VT/Ns and VTS, including legal scope of practice and appropriate delegation of tasks in the clinical setting. Educating currently practicing veterinarians about the importance of these factors is important to change the culture. One way to facilitate this could be through the development of communication templates centered around such topics as identifying/educating about VT/Ns’ skills and knowledge (including self‐advocacy), creating a culture of trust, building a scope of reciprocal support, respect, and autonomy, managing change, promoting interprofessional communication with an emphasis on civility and psychological safety, providing formal training in professionalism, and supporting teambuilding. Looking toward the future, veterinary medicine training institutions could prioritize the employment of credentialed VT/Ns, including VTS, as clinical instructors or lecturers in addition to clinical and administrative roles within their teaching hospitals. An additional solution is to include didactic instruction about the roles, education, and training of all members of the veterinary health care team in the curriculum of veterinary professional programs. Such a focus would enable AVMA Council on Education–accredited programs to guide veterinary students into best practices for task delegation at a crucial stage in their careers.

Extrinsic rewards (e.g., compensation) are received for performing job tasks [40], and concerns about salary are prevalent in the field. The 2022 NAVTA Demographic survey reported that low salary is seen as the most challenging aspect of VT/Ns’ jobs [14]. Although the NAVTA survey found credentialed VT/Ns earn an average of $8.20 more per hour than veterinary assistants ($26.80 vs. $18.60), it appears that many VT/Ns do not perceive a noticeable difference. For example, only 9% of VT/Ns in the NAVTA survey reported feeling that VT/Ns with a bachelor's degree earn more than those with an associate's degree. In time, clearly differentiating roles within practices by task delegation and compliance with VT/N title protection and the scope of practice legislated by state veterinary practice acts may alter the perception that wage competition exists between veterinary assistants and credentialed VT/Ns. Historically, credentialed VT/Ns may have been perceived as more expensive to hire and pay, but the costs incurred by noncredentialled staff turnover may far exceed those associated with employing credentialed VT/Ns. Conservative estimates for the cost of turnover are 50%–60% of the employee's annual salary per employee, with some ranges extending to 90% [41]. Exacerbating the issue of low wages, more than one third of VT/Ns have student loan debt, with an average amount of $29,700 [14].

In addition to making changes within currently operating veterinary hospitals, a relatively untapped opportunity to significantly improve VT/Ns’ utilization, increase their job fulfillment, and decrease their burnout lies within interprofessional education (IPE) [42]. Interprofessional teamwork is vitally important in all healthcare sectors, including veterinary medicine [43, 44]. IPE provides opportunities for students to learn with, about, and from each other in order to address potential misunderstandings of one another's motivations, overcome hierarchic attitudes and professional stereotypes, and clarify overlapping roles and responsibilities [45, 46, 47]. As veterinary schools modify their curricula to best meet the changing needs of the field, it is suggested that opportunities for IPE be imbedded within the program. In order for new veterinary schools in development to meet the growing care demands of the future, it is suggested that IPE be considered while clinical experiences are being structured. IPE experiences can vary greatly, including 1‐day events, guest speakers, small group activities, or full inclusion into the curriculum [42]. Regardless of the format, IPE offers an opportunity for both future veterinarians and VT/Ns to practice the art of interprofessional teamwork, helping to improve job satisfaction while also enhancing animal care. It is likely that an improvement in professional satisfaction for veterinarians will be seen with more efficient utilization of credentialed VT/Ns.

It is important to note the industry‐wide discussion surrounding titles and their semantics when considering this study's limitations. The AVMA defines “veterinary technician” as an individual who has completed a 2‐ or 4‐year degree in veterinary technology, passed a national credentialing examination, and met continuing education and other requirements to receive and maintain a state‐level credential (certificate, registration, or license) [48]. The title “veterinary technician” has historically been indiscriminately applied to both formally educated and credential‐holding individuals and those trained on the job, although many professional associations advocate that the title be reserved at a minimum for credentialed individuals even if they do not hold a degree. In an effort to capture a diversity of perspectives, this survey reflects the prevalence of the term “veterinary technician” by including information about, and responses from, individuals who identify as uncredentialed VTs, in addition to VT/Ns and veterinary assistants, including individuals who may work in states that do not mandate education or credentialing. Any perceived lack of clarity in results due to this decision implies an overall need by the industry to focus on the intentional and consistent use of titles when referring to nondoctor labor in veterinary medicine. For ease of analysis, responses and trends from each group (VT/N credentialed, VT/N not credentialed, veterinary assistants, and VTS) are included separately.

NAVTA and many state boards of veterinary medicine employ similar definitions when establishing title protection and scope of practice guidance; however, alternate pathways to state credentialing for VTs still exist outside of degree completion. For this reason, research and demographic information surrounding VT/Ns are not always specific about the education and credential status of subjects. “Veterinary assistant” is used by these entities to refer to individuals who have been trained on the job or completed a certificate in veterinary assisting without a degree in veterinary technology or another VT credential. There is movement within the industry to adopt the abbreviation “CrVT” to denote “credentialed veterinary technician(s)” in research and public relations in an effort to counteract a tendency to misuse the title “CVT” to denote “credentialed VT” rather than “Certified Veterinary Technician.”

Other limitations of the current study include those inherent in online surveys. Our sample consisted of a small percentage of veterinary professionals as well as a disproportionate number of participants working in emergency and critical care; therefore, caution is warranted when generalizing to veterinary professionals working in other settings. Another limitation is response bias, given the small number of responses from all eligible participants. Veterinary assistants, technicians, and nurses who feel strongly about the topics in this study may have been more likely to respond. In conclusion, this study provides a better understanding of the current perceptions and experiences of veterinary professionals and offers insights into what must change to reduce burnout and increase job fulfillment and an individual's intention to remain in the field.

## Author Contributions

**Lori R. Kogan**: conceptualization, data curation, formal analysis, methodology, writing – original draft, writing – review and editing. **Leslie Carter**: conceptualization, methodology, project administration, writing – review and editing. **Kelly Foltz**: conceptualization, methodology, writing – review and editing.

## Conflicts of Interest

The authors declare no conflicts of interest.

## Supporting information




**Supporting Table 1**: Demographics of survey respondents.
**Supporting Table 2**: Correlations of burnout with satisfaction level for the top ten important job characteristics.
**Supporting Table 3**: Results of the multiple linear regression model predicting burnout as a function of satisfaction level with the top ten important job characteristics.
**Supporting Table 4**: Correlations with fulfillment and satisfaction level for the top ten important job characteristics.
**Supporting Table 5**: Results of the multiple linear regression model predicting fulfillment as a function of satisfaction level with the top ten important job characteristics and supervisory role.
**Supporting Table 6**: Correlations with changed interest in remaining in the field with and satisfaction level for the top ten important job characteristics.
**Supporting Table 7**: Results of the ordinal regression model predicting changed interest in remaining in field as a function of satisfaction level with the top ten important job characteristics.

**Supporting Appendix 1**: Perceptions and experiences of VTS.

**Supporting File 1**: Acknowledgements.

## Endnotes

1 Qualtrics.

2 SPSS, version 28.01, IBM.
