# Supplementary material for: Perceptions and Experiences of Veterinary Assistants, Veterinary Technicians/Nurses, and Veterinary Technician Specialists
Source: J Vet Emerg Crit Care (San Antonio). 2025 Oct 21;35(5):521–32. doi: 10.1111/vec.70043 (PMC12614410; doi:10.1111/vec.70043)
Supplement: Supplementary file 1 — Supporting Table 1: Demographics of survey respondents. Supporting Table 2: Correlations of burnout with satisfaction level for the top ten important job characteristics. Supporting Table 3: Results of the multiple linear regression model predicting burnout as a function of satisfaction level with the top ten important job characteristics. Supporting Table 4: Correlations with fulfillment and satisfaction level for the top ten important job characteristics. Supporting Table 5: Results of the multiple linear regression model predicting fulfillment as a function of satisfaction level with the top ten important job characteristics and supervisory role. Supporting Table 6: Correlations with changed interest in remaining in the field with and satisfaction level for the top ten important job characteristics. Supporting Table 7: Results of the ordinal regression model predicting changed interest in remaining in field as a function of satisfaction level with the top ten important job characteristics. [file VEC-35-521-s001.docx]

Supplementary table 1. Demographics of survey respondents

| **Demographics** | **N** | **%** |
| --- | --- | --- |
| **Role (n=2176)** |  |  |
| Veterinary assistant credentialed | 153 | 6 |
| Veterinary assistant credentialed | 9 | <1 |
| Veterinary technician not credentialed | 222 | 9 |
| Veterinary technician credentialed | 1378 | 56 |
| VTS | 414 | 17 |
| **Time in the field (n=1778)** |  |  |
| Up to 5 years | 323 | 18 |
| 6-10 years | 447 | 25 |
| 11-15 years | 323 | 18 |
| 16-20 years | 265 | 15 |
| 21-30 years | 316 | 18 |
| More than 30 years | 104 | 6 |
| **Age (n=1773)** |  |  |
| 18-29 | 413 | 23 |
| 30-39 | 675 | 38 |
| 40-49 | 465 | 26 |
| 50-59 | 174 | 10 |
| 60-69 | 44 | 3 |
| Older than 69 | 2 | <1 |
| **Gender (n=1761)** |  |  |
| Male | 162 | 10 |
| Female | 1580 | 89 |
| Non-binary, nonconforming | 17 | 1 |
| Prefer not to respond | 17 | 1 |
| Other | 2 | <1 |
| **Degree beyond high school (n=1915)** |  |  |
| Associate's Degree, Veterinary Technology (incl. Applied Science/Applied Technology) | 1107 | 58 |
| Other type of Associate's degree | 214 | 11 |
| Bachelor’s Degree, Veterinary Technology | 278 | 15 |
| Other type of Bachelor's degree | 580 | 30 |
| Master’s Degree in Veterinary Science | 36 | 2 |
| Other type of Master's degree | 85 | 4 |
| **Race (n=1776)** |  |  |
| African American or Black | 59 | 3 |
| Asian | 30 | 2 |
| Biracial/multiracial | 54 | 3 |
| Middle Eastern | 7 | <1 |
| Native American/Indigenous | 18 | 1 |
| Native Hawaiian/Pacific Islander | 17 | 1 |
| White/Caucasian | 1503 | 85 |
| Prefer not to respond | 60 | 3 |
| I prefer to self-describe | 28 | 2 |
| **Ethnicity (n=1769)** |  |  |
| Hispanic/Latinx | 180 | 10 |
| Not Hispanic/Latinx | 1495 | 85 |
| Prefer to not respond | 94 | 5 |
| **Country where work/practice (n=1778)** |  |  |
| United States | 1637 | 92 |
| Canada | 78 | 4 |
| Australia | 26 | 2 |
| New Zealand | 11 | 1 |
| United Kingdom | 15 | 1 |
| Other | 11 | 1 |
| **Current place of employment (n=2101)** |  |  |
| ER and specialty | 1009 | 48 |
| ER only | 185 | 9 |
| General Practice with ER | 360 | 17 |
| Research | 31 | 2 |
| Shelter | 13 | 1 |
| Industry (sales, field educator, pet insurance, etc.) | 30 | 1 |
| Academia | 275 | 13 |
| Non-profit | 18 | 1 |
| Self-employed (consultant) | 18 | 1 |
| Not currently employed | 20 | 1 |
| Other | 142 | 7 |
| **Supervisory role (n=2171)** |  |  |
| Yes | 762 | 35 |
| No | 1409 | 65 |
| **Practice ownership structure (n=1669)** |  |  |
| Corporation (partial or majority-owned) | 1123 | 67 |
| Privately owned | 440 | 26 |
| Other | 78 | 5 |
| I don’t know | 28 | 2 |
| **Place of employment a VECCS-Certified facility (n=1669)** |  |  |
| Yes | 558 | 24 |
| No | 923 | 40 |
| I don't know | 188 | 36 |

Supplemental Table 2. Correlations of burnout with satisfaction level for the top ten important job characteristics

| Pay/salary (N = 1783) | Pearson Correlation | -.280^**^ |
| --- | --- | --- |
|  | Sig. (2-tailed) | <.001 |
| Your ability to contribute to animal well-being (N = 1784) | Pearson Correlation | -.323^**^ |
|  | Sig. (2-tailed) | <.001 |
| Sense of competency (N = 1783) | Pearson Correlation | -.286^**^ |
|  | Sig. (2-tailed) | <.001 |
| Amount/number of staff for optimal patient care (N = 1784) | Pearson Correlation | -.292^**^ |
|  | Sig. (2-tailed) | <.001 |
| Workplace environment (N = 1784) | Pearson Correlation | -.432^**^ |
|  | Sig. (2-tailed) | <.001 |
| Appropriate utilization of your professional skills (N = 1783) | Pearson Correlation | -.269^**^ |
|  | Sig. (2-tailed) | <.001 |
| Respect at work (N = 1784) | Pearson Correlation | -.399^**^ |
|  | Sig. (2-tailed) | <.001 |
| Support from leadership (N = 1784) | Pearson Correlation | -.347^**^ |
|  | Sig. (2-tailed) | <.001 |
| Ability to affect change when see something could be improved (N = 1784) | Pearson Correlation | -.355^**^ |
|  | Sig. (2-tailed) | <.001 |
| Self-improvement and/or professional development (N = 1784) | Pearson Correlation | -.335^**^ |
|  | Sig. (2-tailed) | <.001 |

Supplemental Table 3. Results of the multiple linear regression model predicting burnout as a function of satisfaction level with the top ten important job characteristics

| **ANOVA** | | | | | | | | | | |  |  |
| --- | --- | --- | --- | --- | --- | --- | --- | --- | --- | --- | --- | --- |
| Model | Sum of Squares | | df | Mean Squares | | F | | Sig. | | |  |  |
| Regression  Total | 298.19  5780.78 | | 10  1781 | 29.82 | | 65.03 | | <0.001 | | |  |  |
| **Coefficients* (Dependent Variable: Job Burnout)** | | | | | | | | | | **95.0% CI** | | |
| Variable | | Coefficient (B) | | | Std. Error | | t | | Sig. | Lower Bound | | Upper Bound |
| Intercept | | 3.361 | | | .083 | | 40.726 | | <.001 | 3.199 | | 3.523 |
| **Respect** | | -.098 | | | .020 | | -4.859 | | **<.001** | -.137 | | -.058 |
| **Contribute** | | -.103 | | | .021 | | -4.880 | | **<.001** | -.144 | | -.062 |
| **Pay** | | -.036 | | | .016 | | -2.281 | | **.023** | -.067 | | -.005 |
| Utilization | | .032 | | | .019 | | 1.685 | | .092 | -.005 | | .069 |
| Competency | | -.030 | | | .021 | | -1.463 | | .144 | -.070 | | .010 |
| Support from leadership | | .017 | | | .020 | | .855 | | .392 | -.022 | | .055 |
| **Workplace** | | -.152 | | | .021 | | -7.166 | | **<.001** | -.194 | | -.110 |
| **Amount staff** | | -.075 | | | .016 | | -4.581 | | **<.001** | -.107 | | -.043 |
| **Self improvement** | | -.063 | | | .020 | | -3.159 | | **.002** | -.102 | | -.024 |
| **Affect change** | | -.040 | | | .019 | | -2.150 | | **.032** | -.077 | | -.004 |

Supplemental Table 4. Correlations with fulfillment and satisfaction level for the top ten important job characteristics

| Pay/salary (N= 1783) | Pearson Correlation | .363^**^ |
| --- | --- | --- |
|  | Sig. (2-tailed) | <.001 |
| Your ability to contribute to animal well-being (N= 1784) | Pearson Correlation | .505^**^ |
|  | Sig. (2-tailed) | <.001 |
| Sense of competency (N= 1783) | Pearson Correlation | .462^**^ |
|  | Sig. (2-tailed) | <.001 |
| Amount/number of staff for optimal patient care (N= 1784) | Pearson Correlation | .302^**^ |
|  | Sig. (2-tailed) | <.001 |
| Workplace environment (N= 1784) | Pearson Correlation | .554^**^ |
|  | Sig. (2-tailed) | <.001 |
| Appropriate utilization of your professional skills (N= 1783) | Pearson Correlation | .507^**^ |
|  | Sig. (2-tailed) | <.001 |
| Respect at work (N= 1784) | Pearson Correlation | .562^**^ |
|  | Sig. (2-tailed) | <.001 |
| Support from leadership (N= 1784) | Pearson Correlation | .517^**^ |
|  | Sig. (2-tailed) | <.001 |
| The ability to affect change when you see something that could be improved/changed (N= 1784) | Pearson Correlation | .511^**^ |
|  | Sig. (2-tailed) | <.001 |
| Self-improvement and/or professional development (N= 1784) | Pearson Correlation | .502^**^ |
|  | Sig. (2-tailed) | <.001 |

Supplemental Table 5. Results of the multiple linear regression model predicting fulfillment as a function of satisfaction level with the top ten important job characteristics and supervisory role

| **ANOVA** | | | | | | | | | |  |  |  |
| --- | --- | --- | --- | --- | --- | --- | --- | --- | --- | --- | --- | --- |
| Model | Sum of Squares | | df | Mean Squares | | F | | Sig. | |  |  |  |
| Regression  Total | 610.19  9741.59 | | 11  1781 | 55.47 | | 175.10 | | <0.001 | |  |  |  |
| **Coefficients* (Dependent Variable: Job Fulfillment)** | | | | | | | | | | | **95.0% CI** | |
| Variable | | Coefficient (B) | | | Std. Error | | t | | Sig. | | Lower Bound | Upper Bound |
| Intercept | | -.422 | | | .069 | | -6.153 | | <.001 | | -.557 | -.288 |
| **Supervisor= yes** | | .074 | | | .029 | | 2.525 | | **.012** | | .016 | .131 |
| Supervisor= no | | 0 | | | . | | . | | **.** | | . | . |
| **Respect** | | .117 | | | .017 | | 6.988 | | **<.001** | | .084 | .150 |
| **Contribute** | | .175 | | | .018 | | 9.984 | | **<.001** | | .141 | .210 |
| Pay | | .023 | | | .013 | | 1.735 | | .083 | | -.003 | .049 |
| **Utilization** | | .084 | | | .016 | | 5.364 | | **<.001** | | .053 | .115 |
| **Competency** | | .075 | | | .017 | | 4.395 | | **<.001** | | .042 | .108 |
| Support from leadership | | .030 | | | .016 | | 1.847 | | .065 | | -.002 | .062 |
| **Workplace** | | .126 | | | .018 | | 7.131 | | **<.001** | | .091 | .160 |
| Amount staff | | .014 | | | .014 | | 1.011 | | .312 | | -.013 | .040 |
| **Self improvement** | | .084 | | | .017 | | 5.057 | | **<.001** | | .052 | .117 |
| **Affect change** | | .055 | | | .016 | | 3.470 | | **<.001** | | .024 | .086 |

Supplemental Table 6. Correlations with changed interest in remaining in the field with and satisfaction level for the top ten important job characteristics

| Pay/salary (N = 1887) | Pearson Correlation | .319^**^ |
| --- | --- | --- |
|  | Sig. (2-tailed) | <.001 |
| Your ability to contribute to animal well-being (N = 1888) | Pearson Correlation | .196^**^ |
|  | Sig. (2-tailed) | <.001 |
| Sense of competency (N = 1886) | Pearson Correlation | .207^**^ |
|  | Sig. (2-tailed) | <.001 |
| Amount/number of staff for optimal patient care (N = 1888) | Pearson Correlation | .205^**^ |
|  | Sig. (2-tailed) | <.001 |
| Workplace environment (N = 1888) | Pearson Correlation | .218^**^ |
|  | Sig. (2-tailed) | <.001 |
| Appropriate utilization of your professional skills (N = 1888) | Pearson Correlation | .226^**^ |
|  | Sig. (2-tailed) | <.001 |
| Respect at work (N = 1889) | Pearson Correlation | .252^**^ |
|  | Sig. (2-tailed) | <.001 |
| Support from leadership (N = 1888) | Pearson Correlation | .233^**^ |
|  | Sig. (2-tailed) | <.001 |
| Ability to affect change when you see something that could be improved/changed (N = 1889) | Pearson Correlation | .273^**^ |
|  | Sig. (2-tailed) | <.001 |
| Self-improvement and/or professional development (N = 1889) | Pearson Correlation | .260^**^ |
|  | Sig. (2-tailed) | <.001 |

Supplemental Table 7. Results of the ordinal regression model predicting changed interest in remaining in field as a function of satisfaction level with the top ten important job characteristics

| **Chi-Square** | | **df** | **Sig.** | |  |  |  |  |  |
| --- | --- | --- | --- | --- | --- | --- | --- | --- | --- |
| **326.36** | | 10 | <.001 | |  |  |  |  |  |
| **Coefficients* (Dependent Variable: change in interest in field)** | | | | | | | | **95.0% CI** | |
| Variable | | | Coefficient (B) | | Std. Error | Wald | Sig. | Lower Bound | Upper Bound |
| **Sat respect** | | | **.146** | | **.064** | **5.213** | **.022** | .021 | .272 |
| **Sat contribute** | | | **.162** | | **.071** | **5.276** | **.022** | .024 | .300 |
| **Sat pay** | | | **.365** | | **.048** | **58.975** | **<.001** | .272 | .458 |
| Sat utilization | | | .035 | | .059 | .353 | .552 | -.080 | .150 |
| Sat competency | | | .094 | | .068 | 1.906 | .167 | -.039 | .227 |
| Sat support from leadership | | | -.056 | | .060 | .867 | .352 | -.173 | .061 |
| Sat workplace | | | .006 | | .065 | .008 | .929 | -.122 | .134 |
| **Sat amount staff** | | | **.129** | | **.049** | **6.925** | **.008** | .033 | .226 |
| **Sat self improvement** | | | **.161** | | **.062** | **6.718** | **.010** | .039 | .283 |
| **Sat affect change** | | | **.160** | | **.057** | **7.785** | **.005** | .048 | .272 |
