# Supplementary material for: Perceptions and Experiences of Veterinary Assistants, Veterinary Technicians/Nurses, and Veterinary Technician Specialists
Source: J Vet Emerg Crit Care (San Antonio). 2025 Oct 21;35(5):521–32. doi: 10.1111/vec.70043 (PMC12614410; doi:10.1111/vec.70043)
Supplement: Supplementary file 2 — Supporting Appendix 1: Perceptions and experiences of VTS. [file VEC-35-521-s003.pdf]

Perceptions and experiences of VTS

My name is Lori Kogan and I am a researcher from Colorado State University in the Clinical Sciences department. I would like to invite you to participate in a study that aims to understand the perceptions and experiences of Veterinary Technician Specialists (VTS). Please help by completing a brief (15-minute) survey about your experiences as a VTS.

Who is eligible: Adult (18 years or older) VTS (currently certified or have been certified in the past).
Your participation in this research is voluntary. If you decide to participate in the study, you may stop the survey and close the website at any time, without penalty. We will not collect your name or personal identifiers. When we report and share the data with others, we will combine the data from all participants. Results of this survey will be used for research purposes. While there are no direct benefits to you, the knowledge we gain will help us with our efforts to improve the experience of all VTS.
All participants will have the opportunity at the end of the survey to enter a raffle for a $50 Amazon gift card.
It is not possible to identify all potential risks in research procedures, but the researcher(s) have taken reasonable safeguards to minimize any known and potential (but unknown) risks.

If you have any questions about the research, please contact Lori Kogan at lori.kogan@colostate.edu. If you have any questions about your rights as a volunteer in this research, contact the contact the CSU IRB at: CSU_IRB@colostate.edu; 970-491-1553.

If you consent to complete this survey, please click “Yes I consent” below, to begin the survey.

◯ Yes, I consent to participating in this survey

◯ No, I do not consent to participating in this survey

For the purposes of this survey, the term "credentialed" Veterinary Technician” (crVT) refers to an individual who has completed a formal course of education and training in veterinary technology, such as an accredited degree program, and who maintains an active credential (certificate, license, registration, etc.) in good standing with their state, province, nation, or locality depending on the regulations governing the practice of veterinary technology in their country, state, or province.

The term “Veterinary Technician Specialist” (VTS) refers to individuals who have successfully completed the application and examination process to become a member in good standing of

one of the NAVTA-approved veterinary technician specialty academies and (when indicated) have recertified their credential in accordance with the policy of their respective academy/academies.

Are you currently a credentialed VTS?

- ○ No
- ○ Yes

Were you ever a credentialed VTS?

- ○ No
- ○ Yes

Why are you no longer a credentialed VTS? (select all that apply)

- ☐ Retired
- ☐ Left the field
- ☐ Didn't meet recertification requirements
- ☐ Didn't see a difference in having a VTS
- ☐ Didn't have an incentive to recertify/wasn't using it
- ☐ Couldn't afford the annual dues
- ☐ Other: ____________________

In which specialty academy(ies) are you (or were you) credentialed as a VTS? (select all that apply)

- ☐ AVECCTN (Academy of Veterinary Emergency & Critical Care Technicians and Nurses)
- ☐ AVTAA (Academy of Veterinary Technicians in Anesthesia and Analgesia)
- ☐ AIMVT (Academy of Internal Medicine Veterinary Technicians)
- ☐ AVTCP (Academy of Veterinary Technicians in Clinical Practice)
- ☐ ALAVTN (Academy of Laboratory Animal Veterinary Technicians and Nurses)
- ☐ AVDT (Academy of Veterinary Dental Technicians)
- ☐ AVZMT (Academy of Veterinary Zoological Medicine Technicians)
- ☐ AVBT (Academy of Veterinary Behavior Technicians)
- ☐ AVCPT (Academy of Veterinary Clinical Pathology Technicians)
- ☐ ADVT (Academy of Dermatology Veterinary Technicians)
- ☐ AEVNT (Academy of Equine Veterinary Nursing Technicians)
- ☐ APRVT (Academy of Physical Rehabilitation Veterinary Technicians)
- ☐ AVNT (Academy of Veterinary Nutrition Technicians)
- ☐ AVOT (Academy of Veterinary Ophthalmic Technicians)
- ☐ AVST (Academy of Veterinary Surgical Technicians)
- ☐ AVTDI (Academy of Veterinary Technicians in Diagnostic Imaging)

How long have you worked in the veterinary field?

▼ 5 or fewer years ... More than 30 years

How long have you been (or were you) a credentialed VTS?

▼ Less than 2 years ... More than 20 years

Do you have a veterinary-related Master’s degree?

- ◯ No
- ◯ Yes, what is that degree called? ______________________________

There are many reasons why people decide to become a VTS. Please indicate how important the following potential reasons were in your decision to become a VTS.

	None/minimal importance	Moderately important	Very important
Desire to expand my knowledge base and skills	○	○	○
Desire to achieve personal/professional satisfaction	○	○	○
Desire to be a more impactful member of a veterinary medical team	○	○	○
Desire to elevate the standard of care in my practice	○	○	○
Desire to be perceived as more competent	○	○	○
Desire to be recognized for my advanced experience, knowledge, and skills	○	○	○
Desire to help advance the profession of veterinary nursing	○	○	○
Desire to have more autonomy at work	○	○	○
Desire for more meaning in my career	○	○	○
Desire to perform more advanced procedures	○	○	○
Desire to be better utilized	○	○	○
Desire to have a greater management/supervisory/administrative role	○	○	○
Desire to have a greater teaching role	○	○	○
Desire for greater respect	○	○	○
Desire for higher pay	○	○	○

Encouragement and mentoring by others	○	○	○
Pressure from peers	○	○	○
Pressure from management/administration	○	○	○

Please list any reasons not listed above that significantly impacted your decision to obtain your VTS credentials:

____

Please indicate your response to the following statements as a result of obtaining your VTS certification:

	Not at all true	Somewhat true	Very true
My knowledge, skills, and patient care improved	○	○	○
Obtaining my VTS contributed to elevating the standard of care at my hospital/clinic	○	○	○
I feel like I'm on a better career path	○	○	○
I feel like a more integral member of the veterinary medical team	○	○	○
I feel more competent	○	○	○
I feel others perceive me as more competent	○	○	○
I feel a sense of personal and professional accomplishment	○	○	○
I have more autonomy at work	○	○	○
I feel a deeper sense of purpose and meaning in my work	○	○	○
I perform more advanced procedures than I did prior to obtaining my VTS	○	○	○
I perform more advanced procedures than crVTs without a VTS at my hospital/practice	○	○	○

I have more autonomy than crVTs without a VTS at my hospital/practice	○	○	○
I feel I am better utilized than before I obtained my VTS	○	○	○
I was promoted	○	○	○

Please indicate your response to the following statements as a result of obtaining your VTS certification:

	Not at all true	Somewhat true	Very true
I was offered a leadership (e.g., admin, management, supervisor) role (or a better role)			
I was offered more teaching opportunities or a training role (or a better role)			
I was offered more teaching/administrative off-clinic time			
In general, I feel I have more recognition/respect for my advanced skills, knowledge, and expertise			
The veterinarians I work with trust my knowledge and skills more than before			
I feel like my peers trust my knowledge and skills more than before			
I fulfill a role or purpose in my practice that is different than crVTs without a VTS			
My self-esteem has increased			
My job satisfaction has increased			
I am invited to provide more input into hospital operation decisions			

I have better critical thinking and problem-solving skills	○	○	○
My job title changed to acknowledge my VTS credential	○	○	○
My job description changed to reflect my VTS credential	○	○	○
I collaborate more with DVMs in creating and executing diagnostic and treatment plans	○	○	○

Please indicate your response to the following statements:

	Not at all true	Somewhat true	Very true
I feel that **veterinarians** I work with understand the VTS role and the value it adds to a veterinary practice	○	○	○
I feel that my **peers** understand the VTS role and the value it adds to a veterinary practice	○	○	○
I feel that **clients** understand the VTS role and the value it adds to a veterinary practice	○	○	○

Did you receive a pay increase after obtaining your VTS?

- ◯ No
- ◯ Yes

By what percentage did your gross pay increase after obtaining your VTS?

- ◯ 2% or less
- ◯ 3-5%
- ◯ 6-10%
- ◯ 11-20%
- ◯ 21-30%
- ◯ 31-40%
- ◯ 41-50%
- ◯ 50% or more
- ◯ Don’t know

Did you receive a monetary bonus after obtaining your VTS?

- ◯ No
- ◯ Yes

Does your hospital/employer pay your annual VTS dues?

- No
- Yes

Prior to obtaining your VTS, did you have production income (or a portion of your wage as production)?

- No
- Yes

Since obtaining your VTS, have you been offered production income (or a portion of your wage as production)?

- No
- Yes

Prior to obtaining your VTS, did you have profit sharing or stock/equity options?

- No
- Yes

Since obtaining your VTS, has your hospital/clinic offered you profit sharing or stock/equity options?

- No
- Yes

What is your current job title?

____

Is there a position or role at your hospital/practice that requires VTS credentials?

- ◯ No
- ◯ Yes

What is that position that requires VTS credentials called?

____

Upon obtaining your VTS, was your accomplishment acknowledged by your practice?

- ◯ No
- ◯ Yes- how? ________________________________

How is your VTS credential currently acknowledged?

- [ ] ⊗ My VTS credential is not acknowledged
- [ ] Name badge
- [ ] Uniform marking
- [ ] Whiteboard/bulletin board
- [ ] Schedule
- [ ] During staff orientation
- [ ] Website or social media
- [ ] Other: ____

Page Break

Please indicate how you feel the following factors impact optimal utilization of VTS.

	No impact/barrier	Minimal barrier	Moderate barrier	Large barrier
DVMs' lack of understanding of what a VTS is	◯	◯	◯	◯
DVMs are unaware of VTS advanced skill/training level	◯	◯	◯	◯
DVMs' lack of trust in VTS advanced skill set	◯	◯	◯	◯
Lack of collaboration between VTS and the DVM	◯	◯	◯	◯
DVMs' fear of liability	◯	◯	◯	◯
DVMs' reluctance to relinquish control	◯	◯	◯	◯
DVMs' fear of change	◯	◯	◯	◯
Lack of role clarification/differentiation between VTS and non-VTS duties/skills within the practice	◯	◯	◯	◯
Culture of the practice limits tasks VTS are allowed to perform	◯	◯	◯	◯
Lack of training/mentoring	◯	◯	◯	◯
Legal restrictions	◯	◯	◯	◯
Lack of self-promotion by VTS	◯	◯	◯	◯

Does your practice/hospital educate clients re: VTS requirements/roles/responsibilities?

- ◯ Yes
- ◯ No
- ◯ Don't know

How does your place of employment educate clients about the education and roles of veterinary technicians/nurses? (select all that apply)

- ☐ Hospital posters
- ☐ Flyers/pamphlets
- ☐ Hospital TV/videos
- ☐ Website or other social media
- ☐ Word of mouth/conversation
- ☐ Other: ____

Would you recommend pursuing a VTS to others?

- ◯ No- why not? ____
- ◯ Yes- why? ____

Since obtaining your VTS, and compared to a crVT, which of the following best describes your level of autonomy (independence) in performing each of the following tasks (within the limits of your state's (or other jurisdiction veterinary practice act), and assuming collaboration with a DVM)?

	I did not do this before VTS credentialing, and I still do not do this	Prior to VTS credentialing, I did this with direct supervision	Prior to VTS credentialing, I did this with no/minimal supervision	I did not do this prior to VTS credentialing, but now as a VTS, I do this with direct supervision	I did not do this prior to VTS credentialing, but now as a VTS, I do this with no/minimal supervision
Generate an initial patient problem list	○	○	○	○	○
Recommend an initial diagnostic plan to the DVM	○	○	○	○	○
Interpret/screen results of diagnostic blood work	○	○	○	○	○
Perform TFAST/AFAST ultrasound	○	○	○	○	○
Initiate and coordinate life-saving treatment in an emergency according to standard protocol	○	○	○	○	○
Perform thoracocentesis	○	○	○	○	○
Perform pericardiocentesis	○	○	○	○	○
Manage dialysis patients	○	○	○	○	○
Design anesthetic plans	○	○	○	○	○

Induce, maintain, and monitor anesthesia	◯	◯	◯	◯	◯
Unblock cats	◯	◯	◯	◯	◯
Manage ventilator pts	◯	◯	◯	◯	◯
Conduct routine recheck and follow up appts with report to DVM	◯	◯	◯	◯	◯
Perform euthanasia	◯	◯	◯	◯	◯

In the next two questions, please provide two examples of the highest level skills you perform as a VTS

Example 1 of the highest level skill you perform as a VTS:

____

Example 2 of the highest level skill you perform as a VTS:

____

Do you picture yourself as a VTS five years from now?

◯ No- why not? ______________________________

◯ Yes

As the veterinary field struggles with ongoing personnel shortages and attrition, solutions ranging from expanding the scope of practice of the veterinary technician/nurse to developing advanced level veterinary practitioners (ALVP) who function like human medicine's nurse practitioners and physician assistants are being discussed and implemented.

Please indicate your agreement level with the following statements.

	Strongly disagree	Somewhat disagree	Neither agree nor disagree	Somewhat agree	Strongly agree
In general, crVT (credentialed veterinary technicians/nurses) are underutilized.					
In general, VTS are underutilized.					
VTS seems like the potential natural step between crVTs and advanced level veterinary practitioners.					
As a VTS, I currently function like an ALVP, with the exception of performing functions that are limited to veterinarians by state practice acts (diagnose, prognose, prescribe, or perform surgery).					
As a VTS, I currently function like an ALVP, and frequently collaborate with veterinarians to					

provide input on diagnosing, prognosing, prescribing, or performing surgery, with the veterinarian having the final sign-off.

Are you interested in becoming an advanced level veterinary practitioner?

- ◯ No
- ◯ Yes

Would you be interested in becoming an advanced level veterinary practitioner if it required obtaining a master’s degree?

- ◯ No
- ◯ Yes
- ◯ Unsure

Please indicate your agreement to the following statement:
If a master’s degree was required to become an advanced level veterinary practitioner, VTS credentialing should count towards the requirements of such a degree.

- ◯ Agree
- ◯ Neutral/no opinion
- ◯ Disagree

Which of the following working relationships with a veterinarian do you envision as most appropriate for an advanced level veterinary practitioner (ALVP) (functions like human medicine's nurse practitioners and physician assistants)?

Please use the following definitions when answering this question:

"Direct supervision" means a licensed veterinarian is readily available on the premises where the patient is being treated and has assumed responsibility for the veterinary care given to the patient by a person working under his or her direction.

"Indirect supervision" means a licensed veterinarian need not be on the premises; has given either written or oral instructions for treatment of the patient; is readily available by telephone or other forms of immediate communication; and has assumed responsibility for the veterinary care given to the patient by a person working under his or her direction.

- ◯ Immediate, direct supervision (veterinarian in visual and audible proximity), hands-on collaboration on every case
- ◯ Close direct supervision, (veterinarian in visual proximity), report findings/plan to veterinarian, collaborate for case sign-off with limited vet check of patient and client
- ◯ Direct supervision (veterinarian readily available on premises), report findings/plan to veterinarian, collaborate for case sign-off with brief veterinarian check of pt/client
- ◯ Indirect supervision (veterinarian available by phone/telehealth), report abnormal findings/plan to veterinarian, collaborative sign-off on complicated cases, veterinarian check only when ALVP/ veterinarian not comfortable; otherwise veterinarian reviews cases daily [or weekly] in bulk
- ◯ Indirect supervision, (veterinarian available by phone), collaborative sign-off on complicated cases, veterinarian check via telehealth if ALVP/veterinarian not comfortable
- ◯ Independent care provider, ALVP/veterinarian have level of trust/standing orders such that only the most complicated of cases are reviewed by veterinarian

Comments about advanced level veterinary practitioners:

____

What is your age?

▼ 18-29 ... Prefer not to respond

How do you identify yourself?

▼ Male ... Other

How do you identify yourself?

- ◯ African American or Black
- ◯ Asian
- ◯ Biracial/multiracial
- ◯ Middle Eastern
- ◯ Native American/Indigenous
- ◯ Native Hawaiian/Pacific Islander
- ◯ White/Caucasian
- ◯ Prefer not to respond
- ◯ I prefer to self-describe: ____

What is your ethnicity?

- ◯ Hispanic/Latinx
- ◯ Not Hispanic/Latinx
- ◯ I prefer to not respond

In what region do you primarily practice?

- ◯ US
- ◯ Canada
- ◯ Australia
- ◯ New Zealand
- ◯ United Kingdom
- ◯ Other: ____

In which state do you currently reside?

▼ Alabama ... I do not reside in the United States

Which of the following best describes your current place of employment (if more than one, please select the one you work at the most):

- ◯ ER and specialty
- ◯ ER only
- ◯ Urgent care
- ◯ General Practice with ER
- ◯ Research
- ◯ Shelter
- ◯ Industry (sales, field educator, pet insurance, etc.)
- ◯ Academia
- ◯ Non-profit
- ◯ Self-employed (consultant)
- ◯ Not currently employed
- ◯ Other: ____

Which of the following best describes the ownership structure in the practice/hospital where you primarily work?

- ◯ Corporation (partial or majority-owned)
- ◯ Privately owned
- ◯ Other
- ◯ I don’t know

Additional comments:

Thank you for your time. When you click on the arrow at the bottom right, your responses will be saved and you will automatically be directed to a new webpage where you can enter your contact information for the $50 Amazon gift card.
