# Supplementary material for: Perceptions and Experiences of Veterinary Assistants, Veterinary Technicians/Nurses, and Veterinary Technician Specialists
Source: J Vet Emerg Crit Care (San Antonio). 2025 Oct 21;35(5):521–32. doi: 10.1111/vec.70043 (PMC12614410; doi:10.1111/vec.70043)
Supplement: Supplementary file 3 — Supporting File 1: Acknowledgements. [file VEC-35-521-s002.docx]

Acknowledgements:

For funding the project and assisting with survey dissemination:

Veterinary Emergency and Critical Care Society (VECCS).

For convening and supporting the Veterinary Technician/Nurse Utilization Taskforce (VeTNUT) VECCS Board of Directors

For their time, perspective, and assistance:

VECCS Veterinary Technician/Nurse Utilization Taskforce (VeTNUT) members:

- Leslie Carter, Chair RVT, MS, VTS(ECC)
- Ken Drobatz, (ACVECC liaison), DVM, MSCE, DACVIM, DACVECC
- Trish Farry, RVN, AVN, VTS(ECC)(A/A), TAA GCHEd
- Kelly Foltz, CVT, LVT, RVT, VTS(ECC)
- Melissa Hulgreen, RVTg, VTS(ECC)
- Marie Kerl, DVM, MPH, MBA, DACVIM (SAIM), DACVECC
- Lori Kogan, Ph.D.
- David Liss, MBA, RVT, CVPM, VTS(ECC)
- Kate Parker, LVT, BSc
- Liz Rozanski, DVM, DACVECC, DACVIM (SAIM)
- Elke Rudloff, DVM, DACVECC
- Kristy Veltri, RVT
- Annie Wayne, (ACVECC liaison), DVM, MPH, DACVECC
- Leslie Wereszczak, MS, LVMT, VTS(ECC)
- Kenichiro Yagi, MS, RVT, VTS (ECC), (SAIM)
- Nicolette Zarday, DVM, MPH

The following professional organizations, specialty academies, and groups were kind enough to disseminate the survey to their members and colleagues:

National Association of Veterinary Technicians in America (NAVTA)

NAVTA’s Committee on Veterinary Technician Specialties (CVTS)

Registered Veterinary Technologists and Technicians of Canada (RVTTC)

Ontario Association of Veterinary Technicians (OAVT)

Other Canadian associations?

Veterinary Nurses Council of Australia (VNCA)

New Zealand Veterinary Nurses Association (NZVNA)

The following State veterinary technician associations (in the US)

AL

CA

GA

MA

NE

NV

NH

PA

SD

TN

UT

WV

MN

NJ

Academy of Veterinary Emergency & Critical Care Technicians and Nurses (AVECCTN)

Academy of Veterinary Technicians in Anesthesia and Analgesia (AVTAA)

Academy of Internal Medicine Veterinary Technicians (AIMVT)

Academy of Physical Rehabilitation Veterinary Technicians (APRVT)

Academy of Veterinary Nutrition Technicians (AVNT) ?

American College of Veterinary Emergency and Critical Care (ACVECC)

American Association of Veterinary Medical Colleges (AAVMC), Hospital Directors

North American Veterinary Community (NAVC)?

VMX?

Veterinary Hospital Managers Association (VHMA)

Blue Pearl Specialty and Emergency Pet Hospitals

Veterinary Emergency Group (VEG)

Vets Now (UK)

Facebook groups:

Credentialed VT Speaker's Bureau

ER Vet Tech Rounds

Vet Tech Café

Chirrups and Chatter

Veterinary Team Training

Vet Tech Life

Veterinary ECC Small Talk

Veterinary Nurse Network

Veterinary Technician Internal Medicine Enthusiasts (VTIME)

Veterinary Technician Management and Discussion Group

Association of Industry Veterinary Technicians

Vet Tech Nation

Feline Veterinary Technicians Unite

Credentialed Veterinary Technicians

Veterinary Technician Specialty Applicants/Candidates

Vet Tech ECG/Cardio Rounds

Listserv to all vet school tech supervisors in the US

Staff at veterinary teaching hospitals associated with the following colleges of Vet Med:

Auburn University

Louisiana State University

University of California Davis

Colorado State University

Tufts University

University of Tennessee

University of Florida

Royal Vet College

Thank you to anyone else whose survey posting confirmation we may have missed.

And a very special thank you to all the veterinary technicians/nurses and assistants who made the effort to complete this survey.
